# Successful treatment of recurrent *Helicobacter fennelliae* bacteraemia by selective digestive decontamination with kanamycin in a lung cancer patient receiving chemotherapy

**DOI:** 10.1099/jmmcr.0.005069

**Published:** 2016-10-31

**Authors:** Yoshihiro Fujiya, Maki Nagamatsu, Junko Tomida, Yoshiaki Kawamura, Kei Yamamoto, Momoko Mawatari, Satoshi Kutsuna, Nozomi Takeshita, Kayoko Hayakawa, Shuzo Kanagawa, Kazuhisa Mezaki, Masao Hashimoto, Satoru Ishii, Norio Ohmagari

**Affiliations:** ^1^​ Disease Control and Prevention Center, National Center for Global Health and Medicine, Tokyo, Japan; ^2^​ Department of Microbiology, School of Pharmacy, Aichi Gakuin University, Aichi, Japan; ^3^​ Department of Clinical Laboratory, National Center for Global Health and Medicine, Tokyo, Japan; ^4^​ Respiratory Medicine, National Center for Global Health and Medicine, Tokyo, Japan

**Keywords:** recurrent bacteremia, lung cancer, chemotherapy, *Helicobacter fennelliae*, selective digestive decontamination, kanamycin

## Abstract

**Introduction:**

*Helicobacter fennelliae* is an enterohepatic *Helicobacter* species causing bacteraemia in immunocompromised hosts. Only a few cases of recurrent *H. fennelliae* bacteraemia have been reported in Japan and there are no guidelines regarding antimicrobial treatment for *H. fennelliae* infection.

**Case presentation:**

*H. fennelliae* bacteraemia was observed in a patient receiving platinum-based chemotherapy for lung cancer. To prevent recurrence, the patient received antibiotic therapy with cefepime, amoxicillin and doxycycline for 6 weeks, which is similar to the therapy for *Helicobacter* *cinaedi* bacteraemia. Bacteraemia recurred despite the long-term antibiotic therapy. We hypothesized that the *H. fennelliae* bacteraemia originated from endogenous infection in the intestinal tract due to the long-term damage of the enteric mucosa by platinum-based drugs and performed selective digestive decontamination (SDD) with kanamycin. Bacteraemia did not recur after SDD.

**Conclusion:**

Our observations indicate that clinicians should be aware of possible recurrent *H. fennelliae* bacteraemia, which could be effectively prevented by SDD with kanamycin.

## Introduction

*Helicobacter cinaedi* and *Helicobacter fennelliae* are spiral-shaped Gram-negative rods that are enterohepatic *Helicobacter* species; they inhabit the colons of humans and animals, and cause bacteraemia in immunocompromised hosts, especially in patients with human immunodeficiency virus or haematological malignancies (Kemper *et al.*, 1993; Kiehlbauch *et al.*, 1995; Ng *et al.*, 1987; Hsueh *et al.*, 1999; Rimbara *et al.*, 2013a, b; Saito *et al.*, 2016). *H. cinaedi* and *H. fennelliae* also cause bacteraemia in patients with chronic renal failure, autoimmune diseases and solid organ cancers (Nishine *et al.*, 2007; Matsumoto *et al.*, 2007; Rimbara *et al.*, 2013b; Saito *et al.*, 2016). There are several reports of recurrent *H. cinaedi* bacteraemia in immunocompromised hosts (Sullivan *et al.*, 1997; Mammen *et al.*, 1995; Kikuchi *et al.*, 2012; Uçkay *et al.*, 2006), which is treated with prolonged antibiotic therapy to prevent further recurrence (Kiehlbauch *et al.*, 1994; Tee *et al.*, 1996; Sullivan *et al.*, 1997). However, only a few cases of recurrent *H. fennelliae* bacteraemia have been reported in Japan (Saito *et al.*, 2016), and there are no guidelines regarding the choice or duration of antimicrobial treatment and prevention of recurrence for *H. fennelliae* infection. Here, we describe a case of recurrent *H. fennelliae* bacteraemia, which occurred in a patient receiving platinum-based chemotherapy for solid organ cancer. The recurrence of *H. fennelliae* bacteraemia in this case was successfully prevented by selective digestive decontamination (SDD) with oral kanamycin.

## Case report

A 73-year-old man suffering from advanced lung adenocarcinoma with multiple metastases was admitted to our hospital in Shinjuku-ku, Tokyo, for a third cycle of chemotherapy. His lung adenocarcinoma diagnosed 8 years previously had gradually progressed despite surgical treatment, chemotherapy and radiation therapy. He also had a medical history of thyroid papillary carcinoma treated by thyroidectomy 7 years previously. The patient had received two cycles of carboplatin/pemetrexed (CBDCA/PEM) therapy within the 4 months prior to this admission.

On hospital day 2, 43 days after the second cycle of chemotherapy, the patient developed a fever (38.3 °C); however, no other symptoms, such as shaking chills, diarrhoea, abdominal pain or extremity pain, were observed. His physical parameters were almost normal. Laboratory examinations showed a white blood cell count of 8640 cells µl^−1^, a neutrophil count of 6790 cells µl^−1^ and a C-reactive protein level of 16.2 mg dl^−1^, indicating a strong inflammatory response without neutropenia. Chest X-ray findings were the same as in previous tests. The patient was treated empirically with ampicillin/sulbactam (3 g twice a day) for 2 days without clinical improvement, and then with cefepime (2 g twice a day), after which the fever gradually subsided.

After the initiation of ampicillin/sulbactam therapy, two sets of aerobic and anaerobic blood cultures were performed using BACTEC Plus Aerobic/F culture vials and BACTEC Plus Anaerobic/F culture vials (Becton, Dickinson), respectively. On hospital day 7, one of the two aerobic cultures became positive after 6 days of incubation in BACTEC 9240 medium (Becton, Dickinson), whereas both anaerobic cultures remained negative. Gram staining of the positive culture revealed spiral-shaped Gram-negative rods. Subsequent subculturing on Nissui sheep blood agar plates and Nissui modified Skirrow’s medium EX plates (Nissui Pharmaceutical) revealed thin film-forming colonies and scanty transparent colonies, respectively, after 7 days of incubation at 35 °C in a microaerobic atmosphere (6–12 % O_2_, 5–8 % CO_2_). *H. cinaedi* was suspected based on the colony characteristics and the biochemical properties of the isolate were tested by the API Campy system (bioMérieux).

**Fig. 1.**
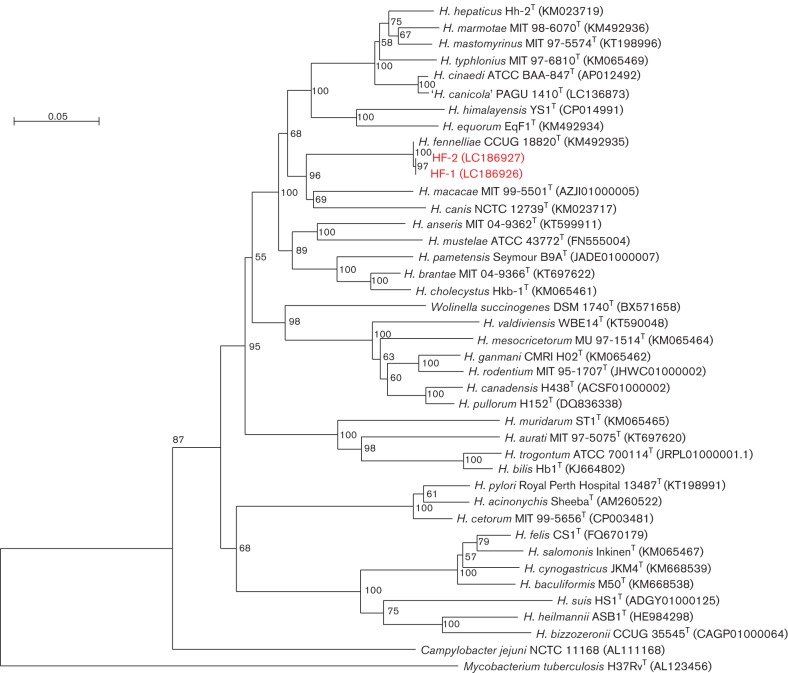
Phylogenetic tree based on the GyrA protein sequences showing the position of our two isolates (HF-1 and HF-2) within the genus *Helicobacter*. GenBank/EMBL/DDBJ accession numbers and type strains are indicated. The numbers at the branching points are bootstrap percentages (based on 1000 replications). The evolutionally distances were computed using the Kimura empirical model, and the phylogenetic tree was constructed using the NJplot software. The bar represents 1 inferred amino acid substitution per 100 amino acids.

The isolate was initially identified as *H. fennelliae* (microcode 4401064) with a relatively low probability level of 80.2 %. However, the biochemical characteristics of nitrate reduction, alkaline phosphatase production and esterase activity were different from those of *H. cinaedi*. To confirm the identification, the *gyrA* gene of the isolate was sequenced (2450 bp) and the deduced protein sequence (811 residues) used to determine the phylogenetic relationship with the GyrA protein of *H. fennelliae* CCUG 18820^T^, whose sequence was obtained from a public database (Ménard *et al.*, 2016; Kawamura *et al.*, 2016). In the phylogenetic analysis based on GyrA sequences, the isolate (HF-1; accession no. LC186926) was identified as *H. fennelliae* (Fig. 1). Antimicrobial susceptibility was determined by the agar dilution method (Rimbara *et al.*, 2012) Our experiments revealed low MICs of ampicillin, cefepime and doxycycline for our *H. fennelliae* isolate; however, the MIC of ciprofloxacin was much higher than that for *H. fennelliae* CCUG 18820^T^ (Table 1), which is consistent with previous findings (Rimbara *et al.*, 2013b).

**Table 1.** MICs of antibiotics for *H. fennelliae* isolated from the lung cancer patient and the CCUG 18820 strain

	MIC (µg ml^−1^) of :										
*H*. *fennelliae* isolate	Ampicillin	Amoxicillin	Cefepime	Imipenem	Kanamycin	Gentamicin	Clarithromycin	Clindamycin	Doxycycline	Minocycline	Ciprofloxacin
HF-1	0.5	na	1	0.031	0.5	0.25	na	0.5	0.25	0.031	32
HF-2	0.5	na	1	0.031	0.5	0.25	na	0.5	0.25	0.031	32
CCUG 18820*	nd	1	nd	0.031	nd	0.5	0.125	nd	nd	0.125	0.5

na, Not attempted; nd, no data.

*Rimbara *et al.* (2013b).

The patient was treated with cefepime for 15 days, and then with the oral antibiotics doxycycline (100 mg twice a day) and amoxicillin (500 mg three times a day) for 24 days. The long-term antibiotic therapy we used was similar to that used for *H. cinaedi*. The patient received the third cycle of chemotherapy on hospital day 15 and was discharged on hospital day 21.

After 6 weeks of oral antibiotic therapy, the patient was admitted again for a fourth cycle of CBDCA/PEM therapy. On day 7 after chemotherapy, he showed a high-grade fever (39.8 °C) and malaise with no other symptoms. Laboratory tests revealed no neutropenia. *H. fennelliae* bacteraemia was considered, and the patient was treated empirically with cefepime after two sets of blood cultures were performed. One aerobic blood culture was positive after 5 days of incubation, demonstrating spiral-shaped Gram-negative rods. Subcultures, phenotypic tests, gene sequence determination and phylogenetic analysis of the GyrA protein were performed as described above. This isolate (HF-2; accession no. LC186927) was identified as *H. fennelliae* and was the same as the HF-1 strain (Fig. 1). HF-2 showed an antimicrobial susceptibility similar to that observed in the HF-1 strain. Antibiotic therapy with cefepime continued for 14 days and the patient defervesced.

*H*. *fennelliae* bacteraemia recurred in spite of the long-term oral antibiotic therapy. The patient was still being treated with CBDCA/PEM and was at risk of recurrence. We assumed that the primary source of blood infection was the gastrointestinal (GI) tract, which is known as a common reservoir for *H. cinaedi* (Imafuku *et al.*, 2016; Uçkay *et al.*, 2006), and prescribed SDD with oral kanamycin monosulfate (500 mg four times a day) for 2 weeks. The patient did not have any further episodes of *H. fennelliae* bacteraemia during chemotherapy after completing the SDD.

## Discussion

Here, we report a case of recurrent *H. fennelliae* bacteraemia during platinum-based chemotherapy. Based on the clinical course observed in the patient, we can draw three important conclusions. First, *H. fennelliae* bacteraemia could recur during platinum-based anticancer chemotherapy. Our patient had advanced lung adenocarcinoma, progressing despite chemotherapy. He developed a fever without neutropenia or any other symptoms 43 days after the second cycle of chemotherapy. The portal of *H. fennelliae* entry and the cause of bacteraemia episodes in our case were unknown. It has been reported that *H. fennelliae* could be recovered from stool cultures (Burnens *et al.*, 1993; Smuts & Lastovica, 2011). CBDCA is a platinum-based anticancer chemotherapeutic agent, which sometimes causes chemotherapy-induced mucositis in the GI tract. In some cases, mucosal inflammation can persist for a long time after chemotherapy because of GI dysfunction due to enteric neuropathy associated with the platinum-based agents (Stojanovska *et al.*, 2015). Therefore, we assumed that the *H. fennelliae* bacteraemia in our case originated from endogenous infection in the GI tract due to damage of the enteric mucosa caused by chemotherapy, and that persisting mucosal damage could result in the recurrence of bacteraemia.

Second, recurrent *H. fennelliae* bacteraemia could be prevented by SDD. We chose cefepime, amoxicillin and doxycycline for the treatment based on previous reports (Rimbara *et al.*, 2013b; Kiehlbauch *et al.*, 1995), and the isolate demonstrated high sensitivity to these antibiotics as evidenced by low MICs (Table 1). It should be noted that the MIC of cefepime for *H. fennelliae* has not been reported previously and that cefepime was clinically effective for our patient. However, *H. fennelliae* bacteraemia recurred despite a 6 week antibiotic therapy. Given that *Helicobacter* spp. had not been isolated in specimens from other patients in the same ward either before or after our patient's admission, we considered it to be a recurrence rather than nosocomial infection. Recurrent bacteraemia caused by ‘*Helicobacter rappini'* (now classified as *Helicobacter bilis*) after antibiotic therapy has been reported in a patient with X-linked (Bruton’s) agammaglobulinaemia. In addition, it was reported that in a case of recurrent *H. cinaedi* bacteraemia, SDD with oral kanamycin monosulfate could prevent recurrence (Imafuku *et al.*, 2016). Given that the MICs of aminoglycosides for *H. fennelliae* are low (Kiehlbauch *et al.*, 1995; Rimbara *et al.*, 2013b; Ng *et al.*, 1987) and that the MIC of kanamycin for our isolate was 0.5 µg ml^−1^, we prescribed oral kanamycin. Following SDD, no side effects, including renal dysfunction, were observed and bacteraemia did not recur despite repeated courses of chemotherapy. As kanamycin is rarely absorbed from the intestinal tract (Hewitt & Finegold, 1958), this observation also indicates that the infection originated from the GI system. Our results suggest that SDD with oral kanamycin can be used to prevent the recurrence of *H. fennelliae* bacteraemia. However, this is, to the best of our knowledge, the first case report of SDD used to treat recurrent *H. fennelliae* bacteraemia and large-scale studies are required to comprehensively investigate the application of kanamycin for SDD in such situations.

Third, fluoroquinolones may be clinically less effective for *H. fennelliae* infection. No standardized antimicrobial susceptibility tests are currently available for *H. cinaedi* and *H. fennelliae*. A recent study has examined a broth microdilution method, which revealed high MICs of fluoroquinolones for *H. cinaedi* isolated in Japan (Tomida *et al.*, 2013). Although ciprofloxacin demonstrated low MICs for *H. fennelliae* CCUG 18820^T^ and another previously described isolate (Hsueh *et al.*, 1999), Rimbara *et al.* (2013b) have reported high MICs of ciprofloxacin for Japanese patients infected with *H. fennelliae*, which is consistent with our case and may be attributed to gene mutations. Although antimicrobial susceptibility breakpoints for *H. fennelliae* are yet to be determined, our isolates may be considered resistant to ciprofloxacin (Mégraud & Lehours, 2007), suggesting that at least in Japanese patients infected with *H. fennelliae*, fluoroquinolones, including ciprofloxacin, should be avoided.

In conclusion, our report describes a case of recurrent *H. fennelliae* bacteraemia in a patient with solid organ cancer undergoing platinum-based chemotherapy. Our observations indicate that clinicians should be aware of possible recurrent *H. fennelliae* bacteraemia, which could be effectively prevented by SDD with kanamycin.

## References

[R1] Burnens A. P. Stanley J. Schaad U. B. Nicolet J.(1993). Novel campylobacter-like organism resembling *Helicobacter fennelliae* isolated from a boy with gastroenteritis and from dogs. J Clin Microbiol 31 1916–1917. 8349774 10.1128/jcm.31.7.1916-1917.1993 PMC265659

[R3] Hewitt W. L. Finegold S. M.(1958). Laboratory studies with kanamycin. Ann N Y Acad 76 122–128. 10.1111/j.1749-6632.1958.tb54698.x 13595499

[R4] Hsueh P. R. Teng L. J. Hung C. C. Chen Y. C. Yang P. C. Ho S. W. Luh K. T.(1999). Septic shock due to *Helicobacter fennelliae* in a non-human immunodeficiency virus-infected heterosexual patient. J Clin Microbiol 37 2084–2086. 10325388 10.1128/jcm.37.6.2084-2086.1999 PMC85042

[R5] Imafuku A. Araoka H. Tanaka K. Marui Y. Sawa N. Ubara Y. Takaichi K. Ishii Y. Tomikawa S.(2016). *Helicobacter cinaedi* bacteremia in four renal transplant patients: clinical features and an important suggestion regarding the route of infection. Transpl Infect Dis 18 132–136. 10.1111/tid.12480 26556588

[R6] Kawamura Y. Tomida J. Miyoshi-Akiyama T. Okamoto T. Narita M. Hashimoto K. Cnockaert M. Vandamme P. Morita Y.(2016). Proposal of *Helicobacter canicola* sp. nov., previously identified as *Helicobacter cinaedi*, isolated from canines. Syst Appl Microbiol 39 307–312. 10.1016/j.syapm.2016.06.004 27381809

[R7] Kemper C. A. Mickelsen P. Morton A. Walton B. Deresinski S. C.(1993). *Helicobacter* (*Campylobacter*) *fennelliae*-like organisms as an important but occult cause of bacteraemia in a patient with AIDS. J Infect 26 97–101. 10.1016/0163-4453(93)97128-K 8454896

[R8] Kiehlbauch J. A. Tauxe R. V. Baker C. N. Wachsmuth I. K.(1994). *Helicobacter cinaedi*-associated bacteremia and cellulitis in immunocompromised patients. Ann Intern Med 121 90–93. 10.7326/0003-4819-121-2-199407150-00002 8017741

[R9] Kiehlbauch J. A. Brenner D. J. Cameron D. N. Steigerwalt A. G. Makowski J. M. Baker C. N. Patton C. M. Wachsmuth I. K.(1995). Genotypic and phenotypic characterization of *Helicobacter cinaedi* and *Helicobacter fennelliae* strains isolated from humans and animals. J Clin Microbiol 33 2940–2947. 8576350 10.1128/jcm.33.11.2940-2947.1995 PMC228611

[R10] Kikuchi H. Asako K. Tansho S. Ueda T. Koshio O. Ubagai T. Asahara M. Kawakami S. Ono Y.(2012). Recurrent *Helicobacter cinaedi* cellulitis and bacteremia in a patient with systemic lupus erythematosus. Intern Med 51 3185–3188. 10.2169/internalmedicine.51.8149 23154730

[R11] Mammen M. P. Aronson N. E. Edenfield W. J. Endy T. P.(1995). Recurrent *Helicobacter cinaedi* bacteremia in a patient infected with human immunodeficiency virus: case report. Clin Infect Dis 21 1055– 1056. 10.1093/clinids/21.4.1055 8645814

[R12] Matsumoto T. Goto M. Murakami H. Tanaka T. Nishiyama H. Ono E. Okada C. Sawabe E. Yagoshi M.(2007). Multicenter study to evaluate bloodstream infection by *Helicobacter cinaedi* in Japan. J Clin Microbiol 45 2853–2857. 10.1128/JCM.00465-07 17596362 PMC2045256

[R13] Mégraud F. Lehours P.(2007). *Helicobacter pylori* detection and antimicrobial susceptibility testing. Clin Microbiol Rev 20 280–322. 10.1128/CMR.00033-06 17428887 PMC1865594

[R14] Ménard A. Buissonnière A. Prouzet-Mauléon V. Sifré E. Mégraud F.(2016). The GyrA encoded gene: a pertinent marker for the phylogenetic revision of *Helicobacter* genus. Syst Appl Microbiol 39 77–87. 10.1016/j.syapm.2015.09.008 26829999

[R15] Ng V. L. Hadley W. K. Fennell C. L. Flores B. M. Stamm W. E.(1987). Successive bacteremias with "*C**ampylobacter cinaedi"* and "*C**ampylobacter fennelliae"* in a bisexual male. J Clin Microbiol 25 2008–2009. 3667922 10.1128/jcm.25.10.2008-2009.1987 PMC269389

[R16] Nishine H. Kasai S. Yoshikawa M. Otsuka Y. Tokuda H.(2007). [A case of recurrent *Helicobacter cinaedi*-associated bacteremia in a small cell lung cancer patient during chemotherapy]. Nihon Kokyuki Gakkai Zasshi 45 26–30 [in Japanese]. 17313023

[R2] Rimbara E. Mori S. Matsui M. Suzuki S. Wachino J. Kawamura Y. Shen Z. Fox J. G. Shibayama K.(2012). Molecular epidemiologic analysis and antimicrobial resistance of *Helicobacter cinaedi* isolated from seven hospitals in Japan. J Clin Microbiol 50 2553–2560. 10.1128/JCM.06810-11 22593597 PMC3421485

[R17] Rimbara E. Matsui M. Mori S. Suzuki S. Suzuki M. Kim H. Sekizuka T. Kuroda M. Shibayama K.(2013a). Draft genome sequence of *Helicobacter fennelliae* strain MRY12-0050, isolated from a bacteremia patient. Genome Announc 1 e00512-13 10.1128/genomeA.00512-13 23929465 PMC3738881

[R18] Rimbara E. Mori S. Kim H. Matsui M. Suzuki S. Takahashi S. Yamamoto S. Mukai M. Shibayama K.(2013b). *Helicobacter cinaedi* and *Helicobacter fennelliae* transmission in a hospital from 2008 to 2012. J Clin Microbiol 51 2439–2442. 10.1128/JCM.01035-13 23658263 PMC3697726

[R19] Saito S. Tsukahara M. Ohkusu K. Kurai H.(2016). *Helicobacter fennelliae* bacteremia: three case reports and literature review. Medicine 95 e3556. 10.1097/MD.0000000000003556 27149471 PMC4863788

[R20] Smuts H. E. Lastovica A. J.(2011). Molecular characterization of the 16S rRNA gene of *Helicobacter fennelliae* isolated from stools and blood cultures from paediatric patients in South Africa. J Pathog 2011 217376. 10.4061/2011/217376 22567323 PMC3335488

[R21] Stojanovska V. Sakkal S. Nurgali K.(2015). Platinum-based chemotherapy: gastrointestinal immunomodulation and enteric nervous system toxicity. Am J Physiol Gastrointest Liver Physiol 308 G223–G232. 10.1152/ajpgi.00212.2014 25501548

[R22] Sullivan A. K. Nelson M. R. Walsh J. Gazzard B. G.(1997). Recurrent *Helicobacter cinaedi* cellulitis and bacteraemia in a patient with HIV Infection. Int J STD AIDS 8 59–60. 10.1258/0956462971918634 9043985

[R23] Tee W. Street A. C. Spelman D. Munckhof W. Mijch A.(1996). *Helicobacter cinaedi* bacteraemia: varied clinical manifestations in three homosexual males. Scand J Infect Dis 28 199–203. 10.3109/00365549609049078 8792493

[R24] Tomida J. Oumi A. Okamoto T. Morita Y. Okayama A. Misawa N. Hayashi T. Akaike T. Kawamura Y.(2013). Comparative evaluation of agar dilution and broth microdilution methods for antibiotic susceptibility testing of *Helicobacter cinaedi*. Microbiol Immunol 57 353–358. 10.1111/1348-0421.12044 23668607

[R25] Uçkay I. Garbino J. Dietrich P. Y. Ninet B. Rohner P. Jacomo V.(2006). Recurrent bacteremia with *Helicobacter cinaedi*: case report and review of the literature. BMC Infect Dis 6 86. 10.1186/1471-2334-6-86 16719920 PMC1482711

